# Determination of selenium-containing species, including nanoparticles, in selenium-enriched *Lingzhi* mushrooms

**DOI:** 10.1007/s00216-023-05031-9

**Published:** 2023-11-21

**Authors:** Kelly L. LeBlanc, Tantima Kumlung, Andrés Suárez Priede, Paramee Kumkrong, Thippaya Junvee, Suladda Deawtong, Jörg Bettmer, María Montes-Bayón, Zoltan Mester

**Affiliations:** 1 National Research Council Canada, 1200 Montreal Road, Ottawa, ON Canada; 2 Thailand Institute of Scientific and Technological Research, 35 Moo 3, Klong 5, Khlong Luang, 12120 Pathum Thani Thailand; 3 Instituto de Investigación Sanitaria Del Principado de Asturias (ISPA), Department of Physical and Analytical Chemistry, Faculty of Chemistry, University of Oviedo, C/Julián Clavería 8, 33006 Oviedo, Spain

**Keywords:** Selenomethionine, Biogenic selenium nanoparticles, Single-particle ICP-MS, *Lingzhi*, Mushrooms

## Abstract

Mushrooms are considered a valuable food source due to their high protein and fibre and low fat content, among the other health benefits of their consumption. Selenium is an essential nutrient and is renowned for its chemo-preventative properties. In this study, batches of selenium-enriched *Lingzhi* mushrooms were prepared by growing mycelium and fruit in substrates containing various concentrations of sodium selenite. The mushroom fruit accumulated low levels of selenium with selenomethionine being the most abundant form in all enriched samples. Conversely, the mycelium showed significant selenium accumulation but relatively low proportions of selenomethionine. The red colour of the selenium-enriched mycelia indicated the probable presence of selenium nanoparticles, which was confirmed by single-particle inductively coupled plasma-mass spectrometry. Mean particle diameters of 90–120 nm were observed, with size distributions of 60–250 nm. Additional analysis with transmission electron microscopy confirmed this size distribution and showed that the biogenic selenium nanoparticles were roughly spherical in shape and contained elemental selenium.

**Graphical Abstract:**

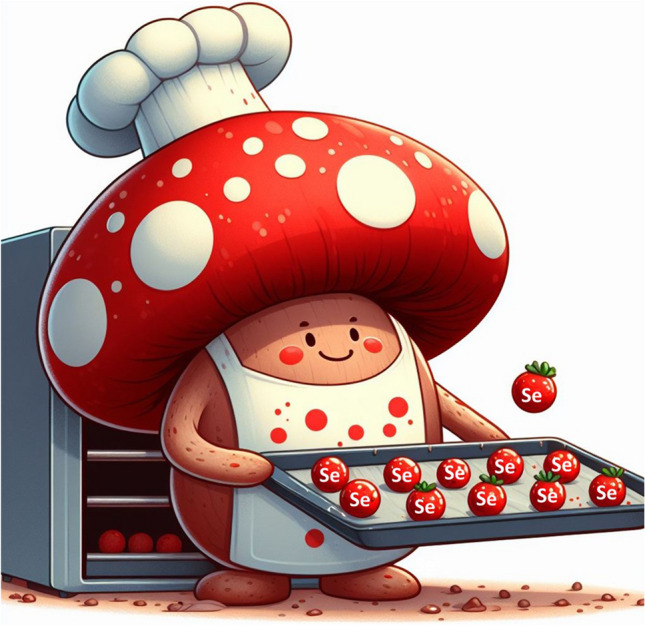

**Supplementary Information:**

The online version contains supplementary material available at 10.1007/s00216-023-05031-9.

## Introduction

*Ganoderma lingzhi* mushrooms, known as *Lingzhi* (Chinese), *het lin chue* (Thai), *Reishi* (Japanese), or *Mannentake* (Korean) [1], are popular items in many Asian households, with the annual market for *Lingzhi*-based products estimated to be more than $2.5 billion USD [2].

The name *Ganoderma lucidum* is frequently used in the literature to refer to *Lingzi*, and is often used interchangeably with *Ganoderma lingzhi* [3]. Despite the fact that Cao et al. [4] have performed phylogenetic analyses demonstrating these species to represent independent lineages, with *G. lingzhi* native to East Asia and *G. lucidum* to Europe and parts of China, the practise of using the names interchangeably still persists [3–5]. Unfortunately, this has caused the information available to become somewhat convoluted—for the purpose of the discussion presented here, we will use the common name *Lingzhi* to refer to all specimens that fall under this umbrella (“*Lingzhi*”, “*Reishi*”, “*G. lucidum*”, and “*G. lingzhi*”), stating the given scientific name where required for clarity.

Mushrooms are considered to be healthy contributions to the diet due to their high protein and fibre and low fat content. Mushrooms are especially valuable in plant-based diets and are rich sources of multi-vitamins and sometimes have other pharmaceutical-like properties. The global mushroom market is projected to grow at an annual rate of 6.74%, increasing from 15.25 million tonnes produced in 2021 to 24.05 million tonnes by 2028; this rate of growth is similar to that of other plant-based foods [6]. *Lingzhi* mushrooms, in particular, have been shown to contain a number of bioactive compounds known to provide various health benefits. Some examples of these are polysaccharides, which are known for their anti-cancer and antimicrobial properties; phenolic compounds, which are known antioxidants and anti-cancer agents; and triterpenoids, which can provide antiviral activity [7].

Many of the above-mentioned nutritional properties are dependent on the substrates on which mushrooms are grown. For example, when grown on selenium-enriched substrates, some mushrooms have higher protein contents compared to control samples [8–11] due to selenium’s affinity to albumins, globulins, and glutelins [8]. Additionally, the production of polysaccharides, which are among the most important bioactive metabolites produced by *G. lingzhi* because of their beneficial effects including immune system promotion and anti-tumor activity [12], can be increased by exposing cultures to relatively low concentrations of selenite (50 mg.kg^−1^). Conversely, elevated (200 mg.kg^−1^) levels of selenite inhibit polysaccharide biosynthesis [13]. There is also evidence that fortification with selenium can help to counteract some of the toxic effects of mercury, which can be accumulated to significant levels by mushrooms when they are grown in contaminated substrates (i.e. due to reduced methylation to methylmercury in the presence of elevated levels of selenium) [14].

The addition of selenium to the substrate during mushroom cultivation also results in the fortification of the mushrooms themselves with selenium, which has a suite of health benefits. The non-uniform distribution of selenium in soils worldwide has resulted in selenium deficiency in the diets of many populations; such deficiencies have been correlated with increased risk of both cancer-related and all-cause mortality [15, 16]. For this reason, many individuals choose to take dietary supplements which include selenium, making the fortification of the element in medicinal *Lingzhi* mushrooms a valuable endeavour.

In a previous study, selenium-enriched *G. lingzhi* was cultivated and resulted in an increased growth rate and spore yield compared to the control experiment. A significant amount of selenium in the growth media was taken up by *G. lingzhi*; however, neither selenite nor other selenium-containing species were identified in the *G. lingzhi* samples [17]. A similar work on *G. lucidum* concluded that growth was not affected by selenium even at high concentrations (200 mg.kg^−1^) and the formation of volatile selenium species, selenomethionine (SeMet) and methylselenocysteine (SeMeCys), was observed; similarly, the red colour of the mycelia grown in that experiment indicated the potential presence of selenium nanoparticles (SeNPs), though this was not confirmed [13]. Therefore, there is still a lack of knowledge on the selenium-containing species that might be formed during selenium enrichment of *Lingzhi* mushrooms.

Here, we grew *G. lingzhi* in two different forms: liquid mycelium cultures and fruiting bodies (the “mushroom” as we traditionally know it). Substrates were fortified with selenium (as selenite) at various concentrations to examine the uptake into the biomass. Since the chemical form of selenium has a significant impact on its bioavailability, with organic forms such as SeMet being more desirable than the inorganic species selenite and selenate [18], speciation experiments were also undertaken. Similarly, because the biotechnological potential of SeNPs looks promising and “green” synthesis of these particles would be advantageous [19, 20], we also performed single-particle analyses to confirm their presence in our cultures. The final aim of the work is to predict which cultivation conditions result in a product with the greatest potential for health benefits if used as dietary supplements.

## Experimental

### Materials for mushroom cultivation

A culture of *G. lingzhi* mycelium was provided by the Expert Center of Innovative Agriculture at the Thailand Institute of Scientific and Technological Research. Yeast malt agar culture medium was purchased from Gibco (Billings, MT, USA). Nutritious liquid medium was prepared from a mixture of yeast malt extract (3.0 g), proteose peptone (5.0 g), and sucrose (10.0 g) in 1 L deionised water (all were AR grade chemicals). Sodium selenite (> 99%) was purchased from Sigma-Aldrich (St. Louis, MO, USA). Sawdust compost (used as a substrate) was prepared from a mixture of para rubber sawdust (100 kg), rice bran (6 kg), calcium hydroxide (1 kg), gypsum (0.5 kg), and magnesium sulphate (0.1 kg); all raw materials were acquired from a local store in Pathum Thani, Thailand.

All instrumentation and liquid media for mycelium culturing, including a 1-L conical flask and 5-L bioreactor (BioFlo ® 310; New Brunswick Scientific GmbH, Nürtingen, Germany), were sterilized by using an autoclave (Astell Scientific Ltd., Sidcup, UK) at 121 °C and 15 PSI for 30 min.

One-kilogram aliquots of sawdust compost spiked with four different concentrations of selenite (to obtain 20, 25, 50, and 100 mg.kg^−1^, prepared by weight disodium selenite and dissolved in deionized water) was transferred into a sterilized grow spawn bag (dimension in cm: 6.5 × 12.5-in. bag) and autoclaved at 121 °C, 15 PSI for 30 min.

### Mycelium cultivation

*G. lingzhi* cultures were dispersed evenly in 1-L conical flasks containing selenite (0, 20, 25, 50, or 100 mg.L^−1^) at a ratio 50 g to 500 mL of liquid media, inoculated for 4 days and transferred to a 5-L bioreactor at 23 ± 5 °C, and stirred at 150 RPM for 5 days; then mycelia were harvested. The mycelium solution was filtered (Whatman GF/C Glass Microfiber Filters 1.2 µm × 47 mm), and the mycelium residue was freeze-dried (Labconco Corporation, Kansas City, MO, USA).

### Mushroom (fruiting body) cultivation

A solution of *G. lingzhi* (without selenite) was inoculated in a compost bag containing sawdust without added selenium for the control, or with various concentrations of selenite (20, 25, 50, and 100 mg.kg^−1^, as described above). The mushrooms were cultivated on shelves in a room 4 m × 6 m × 2.8 m (high) at 25 °C and 60 to 70% relative humidity in the dark for 3 months. After the mushrooms were fully grown, reverse osmosis water was sprayed onto the mushroom fruit twice per day and a fluorescent lamp was applied to the mushrooms for 12 h per day. The harvest was conducted after 20 days of this cycle. The mushrooms were washed with deionized water, chopped, blended, and freeze-dried.

### Determination of total selenium content in mycelium and fruiting body

Approximately 250 mg of dried sample was weighed into a Teflon microwave digestion vessel with 7 mL HNO_3_ and 0.5 mL 30% H_2_O_2_ (w/w). The vessels were capped and the rotor was placed into an Anton Paar Multiwave Pro microwave (Anton Paar; Graz, Austria) and digested by ramping the power to 1400 W over 15 min then holding for 30 min. After a cooling cycle at 0 W, samples were removed from the microwave and gravimetrically diluted to approximately 50 mL with deionized water (DIW).

The digested samples were further diluted to a matrix of approximately 2% HNO_3_ (v/v) and analysed on an Agilent 8800 Triple Quadrupole ICP-MS (Agilent Technologies; Santa Clara, CA, USA) in triple quadrupole mode using O_2_ as cell gas, monitoring the M^+^  > MO^+^ transition for ^74^Se, ^76^Se, ^77^Se, ^78^Se, ^80^Se, and ^82^Se. Solutions of NIST SRM 3149 (selenium standard solution) prepared in 2% HNO_3_ (v/v) were used for external calibration.

### Determination of selenomethionine (SeMet) and water-extractable Se species

For the analysis of SeMet, approximately 250 mg of dried sample and an appropriate volume of a ^82^Se-SeMet standard solution (prepared from NRC CRM SEES-1 [21]), such that the ^80^Se/^82^Se ratio for SeMet was approximately equal to 1 (based on preliminary screening of the samples), were weighted into an Erlenmeyer flask with a ground glass joint. Twenty-four millilitres of 25% methanesulphonic acid and pre-cleaned glass beads were added. The flask was connected to a water-cooled condenser and refluxed on a hotplate for 16 h. Once cooled, the sample was filtered (0.2 µm, PVDF) and stored in the refrigerator until analysis. A sample of NRC CRM SELM-1 (selenized yeast [22]) was also prepared and analysed for quality control. In addition to samples, three blends of SeMet primary standard (from NRC CRM SENS-1 [23]) and spike (^82^Se-SeMet from SEES-1 [21]), and three blanks, were subjected to the same reflux method. Just prior to analysis, samples were diluted 2- to 25-fold with DIW, depending on the expected SeMet concentration.

Samples were analysed for SeMet by HPLC-ICP-MS, where an Agilent 1200 Series HPLC was coupled to an Agilent 8800 Triple Quadrupole ICP-MS (Agilent Technologies, Santa Clara, CA, USA). All samples were analysed using an Agilent Zorbax XDB C18 column, with 100 µL injected for the (diluted) refluxed samples and 25 µL for the aqueous extractions. Mobile phases consisted of 10 mM ammonium formate in DIW adjusted to pH 5.6 and 0.1% formic acid in methanol and were employed in a temperature controlled (40 °C) gradient dilution at 0.4 mL/min: 0–5 min at 5% methanol, ramping to 100% methanol from 5 to 14 min, holding at 100% methanol for 3 min, then re-equilibrating for 6.5 min after a 0.5-min ramp back to 5% methanol. For detection, the ICP-MS was operated in triple quadrupole mode with H_2_ as cell gas, monitoring the M^+^  > M^+^ transition for ^74^Se, ^76^Se, ^77^Se, ^78^Se, ^80^Se, and ^82^Se. To account for the methanol in the mobile phase, the ICP-MS was operated in “organic mode”, using a 1-mm injector, platinum cones, and a brass skimmer base. Oxygen was added to the plasma through the addition of an option gas (20% O_2_ in Ar) at a flow rate set at 15%. The radio frequency (RF) matching was adjusted such that reflected power was minimized. Additionally, a low flow of internal standard (1 mg.kg^−1^ Rh and In) was added to the HPLC eluent, post-column, via a “T” connection.

For quantification of SeMet in the refluxed samples, isotope dilution was employed, following the quadrupole isotope dilution approach, initially described by Pagliano et al. [24] and outlined below:

$${w}_{A}=-\frac{{m}_{{A}^{*}\left(2\right)}{m}_{{A}^{*}\left(3\right)}{r}_{4}+{m}_{{A}^{*}\left(1\right)}{m}_{{A}^{*}\left(3\right)}{r}_{5}+{m}_{{A}^{*}\left(1\right)}{m}_{{A}^{*}\left(2\right)}{r}_{6}}{{m}_{{A}^{*}\left(1\right)}{r}_{4}+{m}_{{A}^{*}\left(2\right)}{r}_{5}+{m}_{{A}^{*}\left(3\right)}{r}_{6}}\bullet \frac{{m}_{B\left(AB\right)}}{{m}_{A\left(AB\right)}}$$

where

$${m}_{{A}^{*}\left(i\right)}={w}_{{A}^{*}\left(i\right)}\bullet \frac{{m}_{{A}^{*}\left({A}^{*}B\left(i\right)\right)}}{{m}_{B\left({A}^{*}B\left(i\right)\right)}}$$

$${r}_{4}=\left({r}_{AB}-{r}_{{A}^{*}B\left(1\right)}\right)\bullet \left({r}_{{A}^{*}B\left(2\right)}-{r}_{{A}^{*}B\left(3\right)}\right)$$

$${r}_{5}=\left({r}_{AB}-{r}_{{A}^{*}B\left(2\right)}\right)\bullet \left({r}_{{A}^{*}B\left(3\right)}-{r}_{{A}^{*}B\left(1\right)}\right)$$

$${r}_{6}=\left({r}_{AB}-{r}_{{A}^{*}B\left(3\right)}\right)\bullet \left({r}_{{A}^{*}B\left(1\right)}-{r}_{{A}^{*}B\left(2\right)}\right)$$

*A*: analyte in sample
A*: analyte in primary standard (natural isotopic composition)
*B*: analyte in isotopically enriched standard
*AB*: mixture of sample and enriched standard
A*B: mixture of primary standard and enriched standard
*w*_X_: mass fraction of X (X = *A*, A*, or* B*)
*m*_X(XY)_: mass of X used to prepare the blend of X and Y (X,Y = *A*, A*, or* B*)
*r*_X_: isotope ratio in X as measured by mass spectrometry (X = *A*, A*, or* B*)

For the analysis of aqueous extractable selenium species, approximately 250 mg of dried sample and 10 mL of DIW were weighed into a 20-mL glass vial. The vials were inverted to mix the contents until the entire sample appeared wet, then they were placed in a sonic bath at room temperature and sonicated for 30 min. After sonication, solids were allowed to settle naturally, then a small amount of supernatant was filtered (0.2 µm, PVDF) for direct analysis.

The aqueous extracts were analysed for inorganic selenium speciation—selenate (Se(IV)) and selenite (Se(VI))—following a second HPLC-ICP-MS procedure. There, a Hamilton PRP-X100 anion exchange column was used, and a gradient elution at 0.8 mL/min and 40 °C was employed using DIW and 200 mM ammonium acetate/200 mM acetic acid, as follows: 2 mM eluent from 0 to 5 min, ramping to 200 mM from 5 to 15 min then holding until 23 min, with a re-equilibration at 2 mM until 30 min. The injection volume was 25 µL and the ICP-MS was operated in triple quadrupole mode with H_2_ as described above, but in normal mode with nickel cones, a 2.5-mm injector, and without the addition of oxygen to the plasma.

For quantification of the unidentified species in the aqueous extracts, their peak areas were compared to that of a standard of known concentration (selenate or selenite) for anion exchange, or SeMet for C18, accounting for differences in sensitivity at various times in the elution profile for the C18 separation. ICP-MS sensitivity is affected by the concentration of methanol in the eluent, so an initial step was taken, which involved running the gradient elution but replacing the internal standard solution with one containing 10 mg.kg^−1^ selenium such that there was a continuous flow of selenium to the ICP-MS. Sensitivity factors were calculated by comparing the signal at all time points (where peaks occurred in the samples) to that during the elution window for SeMet. Based on these factors, concentrations of all unidentified species could be estimated based on a single SeMet standard. This quantification approach has been previously used in our laboratory [25] and has been described in detail by Amayo et al. [26].

### Analysis of selenium nanoparticles (SeNPs)

For the extraction of the SeNPs from the freeze-dried material of *G. lingzhi*, a mechanical lysis protocol was adapted from the one described by R. Álvarez-Fernández et al*.* [27] for SeNP extraction from yeast cells. For this, approximately 15 mg of the dried samples (fruiting bodies and mycelia) was suspended in water (1 mL) and approximately 850 mg of 500-μm-diameter glass beads were added to the suspension. Sample suspensions were placed in an ultrasonic bath for 10 min (Ultrasons, J.P. Selecta S.A., Abrea, Spain), followed by 5 min at maximum speed in a Vortex mixer (Vortex ZX3 VELP Scientifica, Usmate, Italy). This process was carried out twice. After removal of the glass beads, fungi lysates were centrifuged at 300 × *g* for 5 min to remove any tissue debris. Supernatants were collected and diluted with water (dilution 1:2000), prior to the analysis by single-particle ICP-MS (SP-ICP-MS). Earlier experiments revealed that this process could maintain the sizes and size distribution of Se nanoparticles and did not induce any agglomeration [27, 28].

All the SP-ICP-MS measurements were performed on the iCAP™ TQ ICP-MS (Thermo Fisher Scientific, Bremen, Germany), fitted with the “Single-Cell Sample Introduction System” (SC-SIS) (Glass Expansion, Melbourne, Victoria, Australia). Samples were introduced into the equipment at a flow rate of 10 μL.min^−1^ using a syringe pump Chemyx F100X (Chemyx Inc., Stafford, TX, USA) together with a 1-mL Hamilton syringe (Hamilton, Reno, NV, USA). Data acquisition was performed in triple quadrupole mode employing oxygen as reaction gas, monitoring the signals of ^80^Se^+^  > ^80^Se^16^O^+^ and ^31^P^+^  > ^31^P^16^O^+^ for the measurement of ^80^Se^+^ and ^31^P^+^, respectively. The measurement of phosphorous served to prove the successful cell lysis [27]. A dwell time of 5 ms was used. Detailed instrumental conditions are given in Table S1 and Álvarez-Fernández et al*.* [27].

For further data treatment, an established iterative procedure described by F. Laborda et al. [29] was followed. Using this method, the whole data set is averaged and the points above a threshold established at 5σ of the mean are collected as events and extracted from the data set for subsequent iterations. The process is repeated in an iterative way until the number of the detected events remains constant. The limit for possible outliers (multiple-cell events) was set at 3σ above the mean of the resulting data.

For the determination of the selenium mass in individual nanoparticles, an external calibration curve was built analysing ionic selenium solutions in the range 0–50 µg.L^−1^ prepared from an initial 1 g.L^−1^ selenium standard solution (Sigma-Aldrich, St. Louis, MO, USA). Transport efficiency was daily calculated using the gold nanoparticles (AuNPs) quality control material LGCQC5050 (LGC Standards Ltd., Teddington, UK).

Detected SeNPs further underwent a selective reaction for checking the presence of elemental Se (Se^0^) using sodium sulphite. In this reaction, Se^0^ transforms into soluble selenosulphate as described in the literature [30]. For this, 200 µL of fungi lysate was mixed with 200 µL of sodium sulphite solution (0.5 mol.L^−1^, Sigma-Aldrich, St. Louis, MO, USA). The mixture was heated to 50 °C and left to react for 20 min. After cooling down to room temperature, the solution was diluted with water (dilution 1:1000) and analysed by SP-ICP-MS.

As complementary tool, electron transmission microscopy (TEM) (JEOL-2000 21000F, Tokyo, Japan) was employed and the diameters of the detected SeNPs were manually determined employing the open software ImageJ (National Institutes of Health, Bethesda, MD, USA).

## Results and discussion

### Growth of *Lingzhi* mushrooms in substrates with different selenium contents

**Fig. 1:**
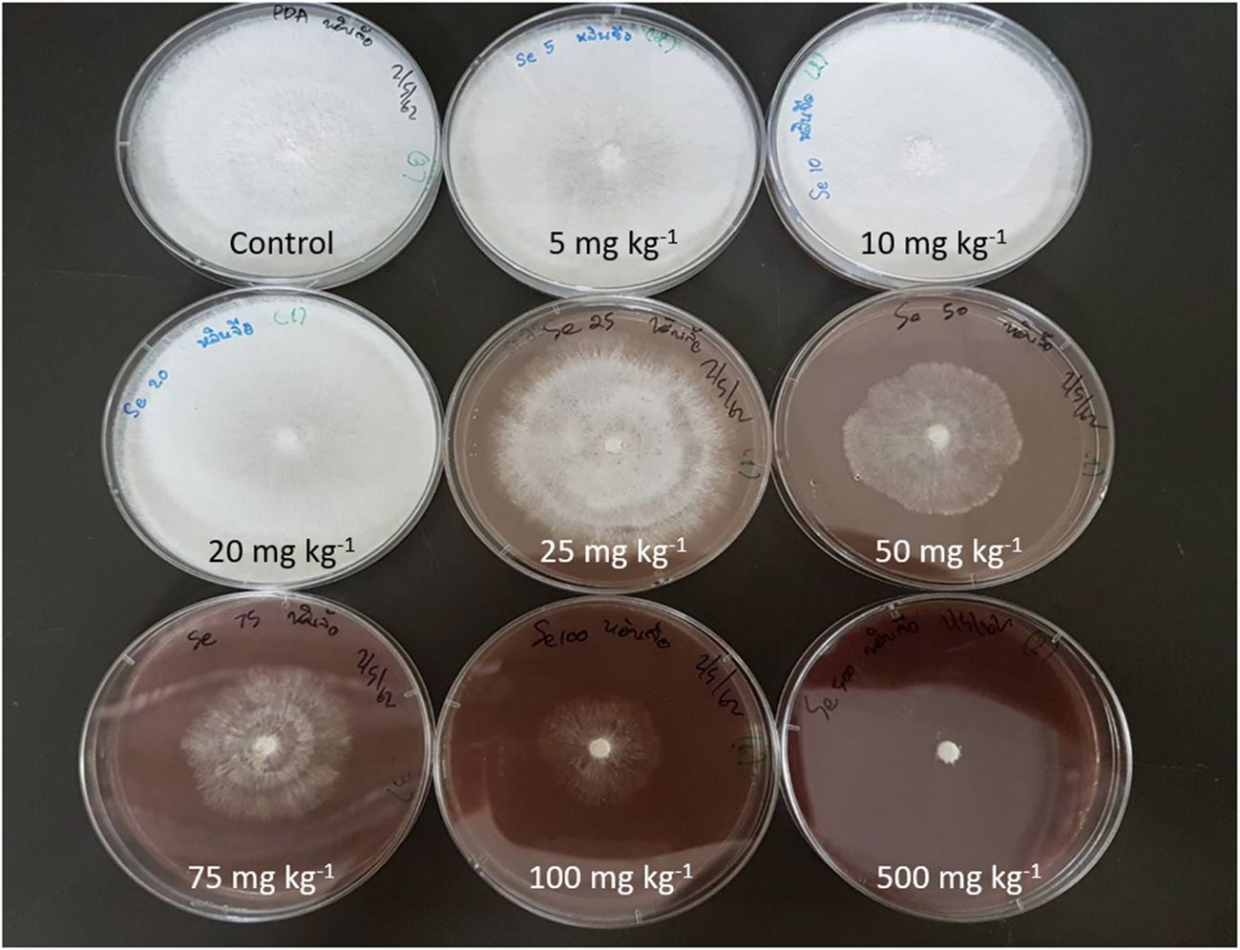
Growth of *G. lingzhi* mycelium in substrates with elevated selenium from the top left to bottom right (0, 5, 10, 20, 25, 50, 75, 100, and 500 mg Se kg^−1^)

*G. lingzhi* mycelium was grown in agar media at various selenium concentrations (0, 5, 10, 20, 25, 50, 100, and 500 mg.kg^−1^). It was noticed that at selenium concentrations of 100 and 500 mg.kg^−1^, the agar colour changed to crimson red after 7 days. The growth of the mycelium was inhibited at these high concentrations as observed by the smallest white spores at the center of the petri dish of the bottom row at the far right in Fig. 1. From the mycelium experiment, the five concentrations of 0, 20, 25, 50, 75, and 100 mg.kg^−1^ were chosen for fruiting body based on the ideal range of concentrations and good biomass production in the mycelium cultures.

**Fig. 2:**
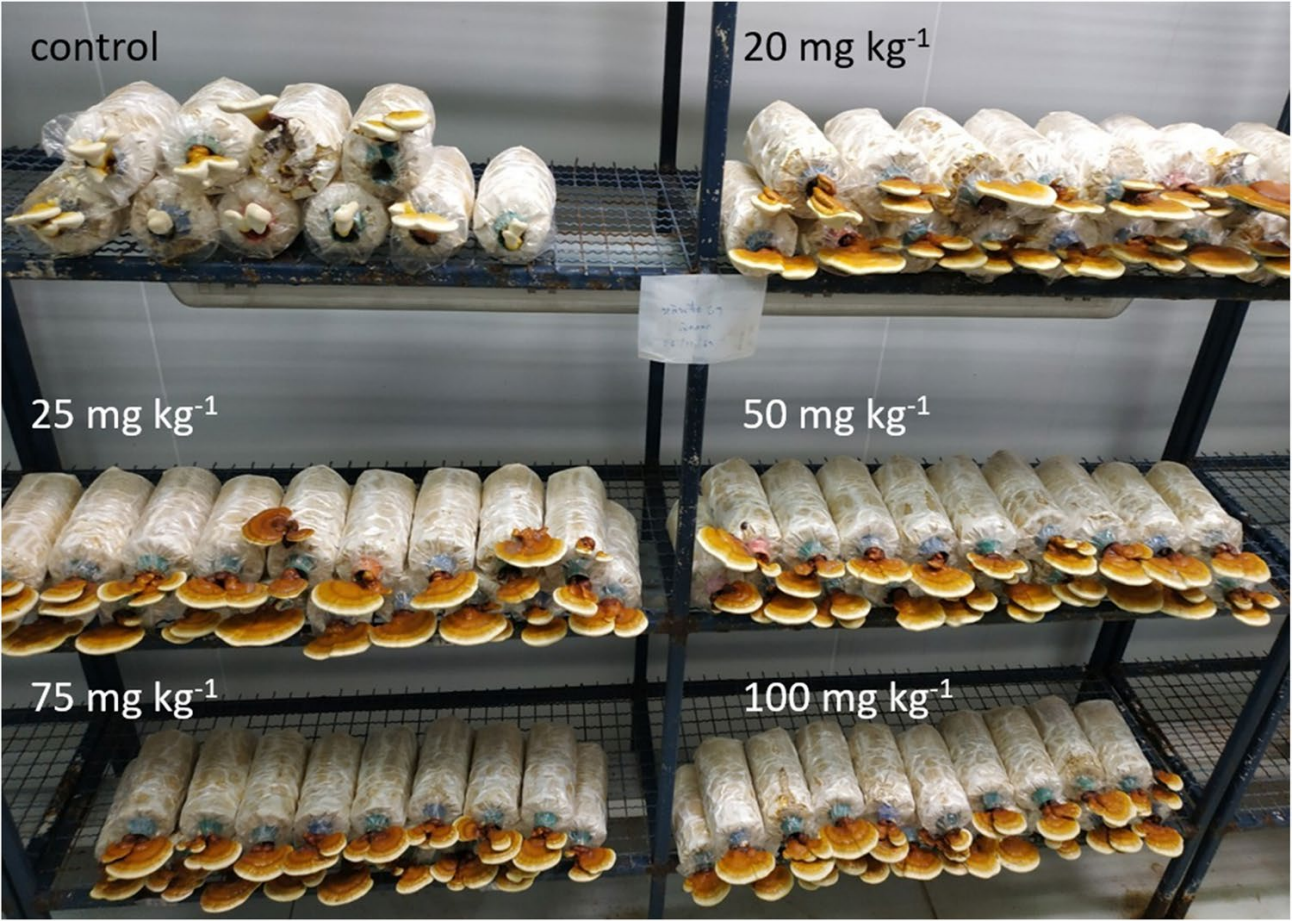
Growth of *G. lingzhi* fruiting bodies in substrates with elevated selenium after 25 days: control mushrooms (no Se), 20 mg Se kg^−1^, 25 mg Se kg^−1^, 50 mg Se kg^−1^, 75 mg Se kg^−1^, and 100 mg Se kg^−1^

When the mycelium was grown in a compost bag enriched with selenium to produce fruiting bodies, no negative visual effects were observed on the growth of mushrooms exposed to elevated selenium concentrations (Fig. S1). Interestingly, it was observed that selenium-enriched mushrooms grew faster and produced a better yield (267–330 g per fresh flower) compared to the control bag (without selenium, 275–280 g per fresh flower) after 25 days of cultivation (Fig. 2).

### Selenium content in mycelium and fruit

#### Total selenium

The total selenium analysis showed low concentrations in the control mycelium and fruiting body samples, but significant accumulation in all selenium-exposed samples (Table 1). Interestingly, the mycelium exposed to the highest amounts (75 mg.kg^−1^) of selenium during growth did not accumulate the largest amount, potentially due to a toxicity threshold being reached. In this study, total selenium content in the mycelium exposed to 50 mg Se kg^−1^ as selenite was 10 × higher than in a similar experiment conducted by Xu et al. [13]. In our work, a lower selenium content was observed in the 75 mg Se kg^−1^ exposure group mycelium, possibly indicating that selenium volatilization occurred as a detoxification mechanism. Conversely, Xu et al. [13] noted nearly 100 × higher total selenium content in *G. lucidum* mycelium exposed to 200 mg Se kg^−1^ (as sodium selenite) than in their cultures exposed to 50 mg Se kg^−1^.

**Table 1:** Total selenium content of *Lingzhi* mushroom samples

	Total selenium content (mg.kg^−1^)	
	Mycelium	Fruiting body
Control*	1.59 ± 0.13	0.136 ± 0.005
20 mg.kg^−1^ Se exposure	204 ± 13	0.890 ± 0.045
25 mg.kg^−1^ Se exposure	435 ± 17	0.430 ± 0.043
50 mg.kg^−1^ Se exposure	4291 ± 95	1.02 ± 0.01
75 or 100 mg.kg^−1^Se exposure**	1731 ± 53	2.74 ± 0.21

*Yeast extract (substrate for mycelium) contained 0.198 ± 0.036 mg Se kg^−1^; sawdust compost (substrate for fruit) contained 0.49 ± 0.05 mg Se kg^−1^

**75 mg.kg^−1^for mycelium, 100 mg.kg^−1^ for fruiting body

In contrast to the mycelia, the mushroom’s fruiting bodies exposed to the highest amounts of selenium also accumulated the element to the greatest extent. It is possible that this could be due to a different selenium transport mechanism being activated when higher selenium concentrations are reached in the biomass.

The total selenium content observed in these mycelium samples are on the same scale as those seen in selenized yeasts, which play an important role in the world of nutritional supplements [18, 31]. Considering the global average recommended daily intake of 60 µg selenium per day (for men; 53 µg per day for women) [15], 14 mg of mycelium from the 50 mg.kg^−1^ exposure group would be required to obtain this intake. It would be quite feasible to provide this in a capsule either by itself or with other materials to make a multi-vitamin type supplement. Conversely, to obtain this from the fruit would require 59 g (dry weight) from the 50 mg.kg^−1^ exposure group. However, in traditional Chinese medicine, *Lingzhi* mushrooms are typically consumed as teas or tinctures, where 3–5 g is steeped for a suggested daily regimen [1]. Assuming the “dregs” are also consumed, this would provide about 8.5% of the recommended daily selenium intake.

#### Selenium speciation

The absolute amount of selenium in a food source or nutritional supplement is only a small part of the overall picture: the true indicator of risk in terms of selenium deficiency (or toxicity) is displayed by an individual’s selenium status which is measured by serum selenium concentrations [15, 16]. The ability of a food or supplement to improve an individual’s selenium status is strongly dependent on the species of selenium present, with organic forms such as SeMet being more desirable than inorganic species such as selenate and selenite [32]. This is also the reasoning behind supplementing the cultures with Se(IV) rather than Se(VI), which is more commonly used: the goal was to produce biologically important reduced Se species and Se(IV) is the reduced inorganic form of selenium and has previously been shown to be taken up by microorganisms [33, 34].

**Fig. 3:**
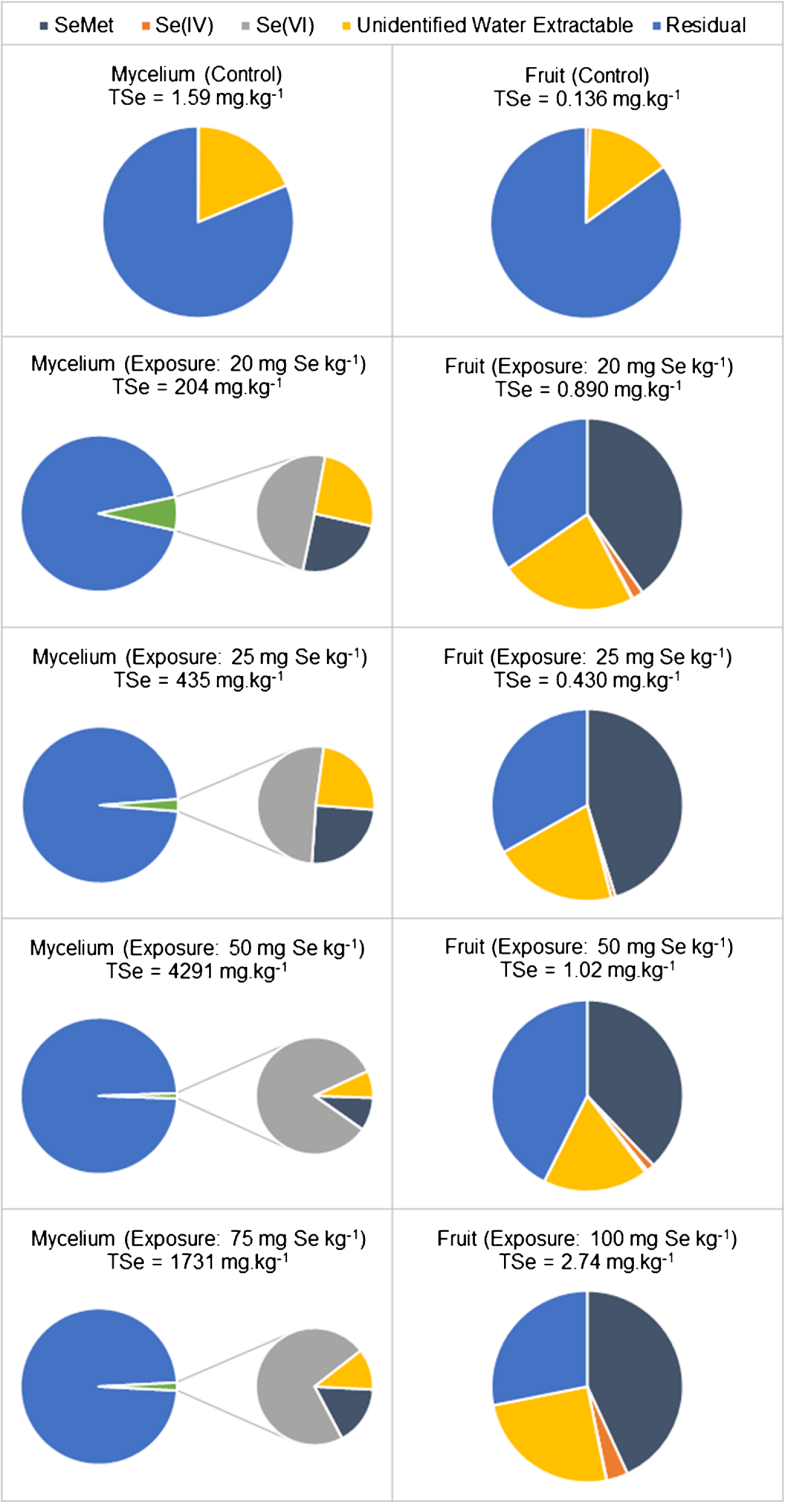
Distribution of (extractable) selenium species measured in *Lingzhi* mushroom samples; note that “fruit” represents the entire fruiting body, lyophilised, ground, homogenized, and sub-sampled

After water extraction (or reflux for recovery of SeMet), the plots on the right in Fig. 3 show the distribution of selenium species in the *Lingzhi* fruiting bodies following exposure to various concentrations of selenite. Despite variation in the total selenium content of the mushrooms, the species distribution remains fairly consistent for all four of the analysed samples. SeMet accounted for a large proportion (38–45%) of the total selenium and it could not be detected in the control samples. Unidentified species in the water extracts accounted for 14–25% of the total selenium in the mushroom fruiting bodies.

In a similar experiment, *G. lingzhi* exposed to selenium at 20–140 mg.kg^−1^ (as selenite) during solid cultivation resulted in significantly higher bioconcentration than observed here, to 127 mg.kg^−1^ total selenium content in the fruiting body [35]. Conversely, when Zhao et al. [36] exposed *G. lucidum* to selenite at 100 mg Se kg^−1^ in the substrate, the resulting total selenium content of the fruiting body was 27.91 ± 0.38 mg.kg^−1^ (of which 56.9 ± 0.3% was determined to be protein-bound). In that work, 18.3% of the protein-bound selenium (about 10.4% of the total selenium) in the 100 mg.kg^−1^ exposed mushrooms was SeMet, and this proportion decreased with increasing selenium exposure (note that the fraction of the total selenium which was protein-bound remained fairly similar between all exposure cultures, varying by only a few percent, but total selenium content increased with increasing selenium in the growth substrate) [36]. Therefore, as the total selenium content of the mushrooms increased, larger fractions were converted to SeMeCys, potentially as an initial metabolic step in a detoxification pathway towards the sequestration of selenium in non-toxic forms [37], similar to the mechanisms observed in hyper-accumulating plant species where SeMeCys is produced [38]. In the present study, a number of unidentified chromatographic peaks were observed in the aqueous extracts from mycelium and fruiting body samples; one of these may be SeMeCys, though further investigation is required to confirm its identity (and then to quantify this species, if present).

An examination of the selenium speciation in the *Lingzhi* mycelia resulted in a very different distribution than that observed for the fruiting bodies (the plots on the left in Fig. 3 show the species distribution in the mycelia). There, the majority of the selenium remained unidentified, with most not being extracted following our protocols (and therefore categorized as “residual”). Moreover, in the water-extractable fraction, it was observed that selenite taken up by the mycelium was completely transformed to selenate (as the major species) along with similar portions of SeMet and an unidentified pool of species. This is an interesting contrast to a similar study [13] where mycelia were grown. There, SeMet accounted for 42.5% of the total selenium in the 50 mg.kg^−1^ exposure group, reaching a maximum on day 6 after increasing in both absolute amount and percent of total selenium. Conversely, that study showed that in the 200 mg.kg^−1^ group, only 8.7% of the total selenium was in the form of SeMet on day 4, with both the absolute and relative amounts of SeMet decreasing, despite significant increases in total selenium content over 3 days (41,574 mg.kg^−1^ on day 6 compared to 1398 mg.kg^−1^ on day 4). Coinciding with this decrease in SeMet was an increase in SeMeCys, which supports the theory that this species is generated as a mechanism of detoxification.

As noted in Fig. 3, a (sometimes significant) fraction of the total selenium present in both the fruiting bodies and the mycelia of the *Lingzhi* mushrooms was categorized as water extractable, but unidentified (not selenate, selenite, or SeMet). HPLC-ICP-MS chromatograms displayed a number of unidentified peaks (see Fig. S2 for an example), indicating an array of selenium-containing metabolites produced by the mushrooms during cultivation (likely similar to the large suite of metabolites produced by yeast when grown in selenium-enriched media [31]). We can begin to speculate on the identity of some of these species based on what has been previously observed for selenized mushrooms. For example, *Pleurotus ostreatus* (oyster mushrooms) grown in the presence of selenite can produce water-soluble seleno-polysaccharides [39]. Further work could be conducted in the future to identify some of the species formed by *G. lingzhi* using techniques such as HPLC with high-resolution molecular mass spectrometry.

Based on the red colour of the mycelium samples, we predicted that much of the “residual” selenium could be actually present in nanoparticulate form. Xu et al. [13] present an excellent image (Fig. 1 in the reference) demonstrating that over time and with increasing selenium exposure, cultures of *G. lucidum* mycelia become increasingly red in colour, suggesting the presence of elemental selenium. Ultra-structural analysis of the mycelia cells (Fig. 2 in the reference) showed a ring of particles outside of the cell (for cultures exposed to 200 mg.kg^−1^ sodium selenite) and the authors suggested that these could be selenium nanoparticles, but did not confirm this hypothesis. Therefore, further efforts were taken here to address the possible presence of nanoparticulate selenium.

#### Selenium nanoparticles (SeNPs)

Several organisms are capable of biogenically producing various nanomaterials following incubation with precursors like metal salts. In the case of SeNPs, several studies have reported their production after exposure to selenite [27, 40–42]. In general, SeNPs might have—among others—antimicrobial and antifungal activities, so that their potential presence in food products has significantly increased a general interest in these selenium species.

With the observed change of colour of the mycelium samples after incubation with selenite, Fig. 1 already indicated the possible presence of selenium in its nanoparticulated form. In order to detect and characterize potentially formed SeNPs, single-particle ICP-MS was employed as described in the “Experimental” section. The most critical step within such an approach was the appropriate sample preparation and the extraction of the sought species. Based on our former work on the detection of SeNPs in yeast [27], a mechanical procedure was applied to the freeze-dried samples (fruiting bodies and mycelia). In order to ensure a complete lysis of the materials, phosphorous was monitored as being a constitutive element in cells. In previous studies, this strategy successfully enabled the detection of nanomaterials in different species of bacteria [43, 44]. The absence of any signals of ^31^P^16^O^+^ in the single-particle ICP-MS experiments was a clear hint for a successful degradation of elongated cells that compose filament structures called hyphae in the mycelium of the mushroom, as observed in all analysed samples of *G. lingzhi*.

**Fig. 4:**
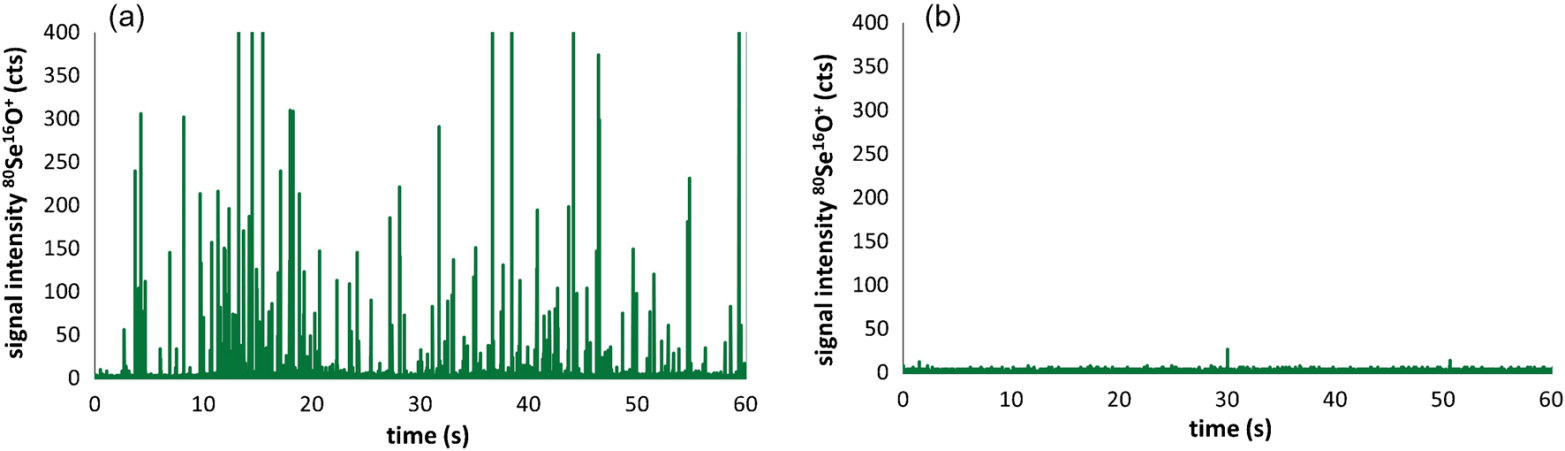
**a** Typical time-resolved single-particle ICP-MS experiment of a mycelium extract after exposure to 50 mg Se kg^−1^ as selenite (indicating SeNP “events”), **b** of the same extract after treatment with sulphite (no SeNP signals)

Figure 4a shows a typical time-resolved measurement of a single-particle ICP-MS experiment of a mycelium after exposure to 50 mg.kg^−1^. The appearance of a number of individual events indicated the presence of selenium-containing nanoparticles (the analysis of the control group did not show any Se-containing events). Similar results were observed for all mycelia, whereas the fruiting bodies of the mushrooms did not reveal any evidence of SeNPs. This observation might be related to the significantly lower concentrations of total selenium in the fruiting bodies (see Table 1) but also to the fact that the mycelium is the more metabolically active part of the mushroom. Single-particle ICP-MS using suspensions with dilution factors much lower than 2000 resulted in an enhanced background signal, but no significant events could be observed for these parts of the mushrooms. This finding might indicate that the transport of nanoparticulate selenium from the mycelium was hampered within the analysed organism.

Additional information about the observed SeNPs could be obtained by the treatment of the extracts with sulphite. In the presence of elemental Se(0), sulphite forms selectively soluble selenosulphate following the reaction: ${\mathrm{Se}}^{0}+{\mathrm{Na}}_{2}{\mathrm{SO}}_{3}\rightleftharpoons {\mathrm{Na}}_{2}{\mathrm{SeSO}}_{3}$

As shown in Fig. 4b, the signals for ^80^Se^16^O^+^ disappeared completely. This clearly indicated that the nanoparticles formed in the mycelia were consisting of elemental Se(0). The required reduction step from Se(IV) to Se(0) was investigated in former studies and depending on the organisms involved, different reductases in the presence of electron donors like nicotinamide adenine dinucleotide (NADH) and others play a crucial role for this process, which can occur differently in the mycelium and in the fruiting body due to their significant metabolic differences [45].

**Fig. 5:**
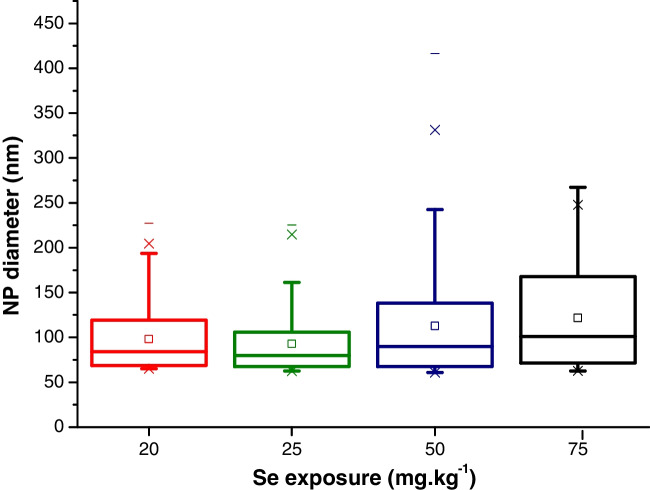
Boxplot charts of the obtained diameters of SeNPs as a function of the exposure concentration of selenite

Further characterization of the SeNP fraction in the mycelium samples was directed to the determination of their sizes and size distributions. This required quantitative evaluation of the time-resolved single-particle ICP-MS experiments including the determination of the transport efficiency and detection sensitivity by an external calibration as described in the experimental part. Under consideration of spherical nanoparticles made of Se(0), Fig. 5 reveals the obtained size variations in function of the selenite concentration used for incubation. The corresponding histograms are shown in Fig. S3.

The mean values of the diameters of the SeNPs did slightly vary between 90 and 120 nm with increasing selenite exposure concentrations, but the observed size distributions (approx. from 60 to 250 nm) did not show statistically significant differences. Under the experimental conditions for this type of sample, the detection limits (spherical elemental SeNPs) were 55 nm in diameter. For the detection of smaller particles, an established HPLC method was applied [27], but none of the samples showed detectable amounts of SeNPs (< 50 nm diameter). As complementary proof, TEM images were recorded. Exemplarily, Fig. S4 represents a mycelium extract after exposure with 50 mg Se kg^−1^ as selenite. Although the number of observed nanoparticles was relatively small, the measured sizes ranged between 60 and 100 nm. This was in accordance with the most abundant SeNPs determined by the ICP-MS analyses (in the range of 60 and 80 nm, Fig. S3). It is noteworthy that all observed SeNPs represented nearly spherical shapes (as in Fig. S4).

The formation of SeNPs in the mycelium is not surprising. In fact, there is a so-called myco-nanotechnological process of NP synthesis, by treating the fungus mycelium with a metal salt solution which causes the fungi to manufacture their metabolites and activate enzymes to survive. The catalytic effect of these extracellular enzymes and metabolites influences the reduction of toxic metal ions from non-toxic metallic solid NPs. In the case of silver NPs, for instance, extracellular fungal reduced nicotinamide adenine dinucleotide (NADH) and NADH-dependent reductases are responsible for Ag^+^ to Ag(0) reduction [46].

Vijayakumar et al. [47] recently summarized the observed size distributions of biogenically produced SeNPs. In regard to those produced biogenically, mainly spherical particles were detected in the size range of 30 to 220 nm. Nanoparticles containing elemental Se(0) produced in the mycelium of the fungus *Phycomyces blakesleeanus* ranged from 32 to 96 nm with an average value of 57 nm [41]. The authors pointed out that further investigations were necessary as the obtained particle dimensions might have great biotechnological potential. The results of this work revealed highly abundant SeNPs in a similar range, so that the mycelium of *G. lingzhi* might be an attractive candidate for the nutritional supplementation of selenium and for further exploration of the beneficial role of biogenically formed SeNPs.

### Significance of the selenium distribution in Lingzhi mushrooms

The fortification of the growth media or substrate with selenium affected the growth of *Lingzhi* mushrooms: as the concentrations of selenite in the media increased, mycelium biomass production decreased, though the opposite effect was noted for the fruiting bodies. Although they were selenium-enriched up to 20-fold compared to the control, total selenium concentrations in the *Lingzhi* fruiting bodies were fairly low compared to the mycelia where up to a 2700-fold increase in selenium content, relative to the control, was noted. In both cases, the resulting selenium species were of biological importance: selenomethionine in the fruit and selenium nanoparticles in the mycelia. Research into the efficiency of SeNPs used as nutritional supplements is ongoing, but the outlook is positive. Therefore, the availability of sustainable sources of SeNPs would be a vital step forward in the natural health products industry. *Lingzhi* mycelia grow quickly and can efficiently reduce high concentrations of selenite to SeNPs. While more research into the biological activity of biogenic SeNPs for this source is still required, combining the health benefits of SeNPs [19, 20] with those of the mushrooms themselves [7], these initial results suggest that *Lingzhi* may be a candidate for selenium supplementation for medicinal use. In future works, we will aim towards a more robust quantitation of biologically relevant selenium species—particularly SeNPs, which were not quantified here—in order to have a better understanding of the relative proportions of both known and yet-to-be-identified selenium species.

### Supplementary Information

Below is the link to the electronic supplementary material.

Supplementary file1 (PDF 426 KB)
