# Supplementary material for: Determination of selenium-containing species, including nanoparticles, in selenium-enriched Lingzhi mushrooms
Source: Anal Bioanal Chem. 2023 Nov 21;416(11):2761–72. doi: 10.1007/s00216-023-05031-9 (PMC11009765; doi:10.1007/s00216-023-05031-9)
Supplement: Supplementary file 1 — Supplementary file1 (PDF 426 KB) [file 216_2023_5031_MOESM1_ESM.pdf]

Supplementary Information for:

Determination of Selenium-Containing Species, Including Nanoparticles, in Selenium-Enriched *Lingzhi* Mushrooms

Kelly L. LeBlanc[1*], Tantima Kumlung[2], Andrés Suárez Priede[3], Paramee Kumkrong[1,2], Thippaya Junvee[2], Suladda Deawtong[2], Jörg Bettmer[3], María Montes-Bayón[3], Zoltan Mester[1]

[1] National Research Council Canada; 1200 Montreal Road, Ottawa, Ontario, Canada

[2] Thailand Institute of Scientific and Technological Research, 35 Moo 3, Klong 5, Khlong Luang, Pathum Thani, 12120, Thailand

[3] University of Oviedo, Faculty of Chemistry, Dept. of Physical and Analytical Chemistry and Instituto de Investigación Sanitaria Del Principado de Asturias (ISPA), C/Julián Clavería 8, E-33006, Oviedo, Spain

* Corresponding Author Email: Kelly.LeBlanc@nrc-cnrc.gc.ca

Contents of the Supplementary Information

Page S2 Table S1: Operating conditions of the iCAP™ TQ ICP-MS for single particle measurements

Page S3 Figure S1: Photos of *G. lingzhi* fruiting bodies of a control mushroom and a mushroom grown in the presence of 100 mg Se kg^{-1} substrate

Page S4 Figure S2: HPLC-ICP-MS chromatogram (C18 column) of a water extract from a sample of Lingzhi mycelium exposed to 20 mg Se kg^{-1} during growth

Figure S3: Histograms of the size distribution obtained by single particle ICP-MS in function of the concentration of selenite used for exposure

Page S5 Figure S4: Transmission electron microscopy image of a mycelium extract after exposure to 50 mg Se kg^{-1} as selenite

References

Table S1: Operating conditions of the iCAP^{TM} TQ ICP-MS for single particle measurements

**Parameter**	**Value**
Coolant gas flow (Ar) (L/min)	14
Auxiliary gas flow (Ar) (L/min)	0.8
Carrier gas flow (Ar) (L/min)	0.52
Sheath gas flow (Ar) (L/min)	0.48
Sample flow rate (µL/min)	10
Dwell time (s)	0.005
Measurement time (s)	120
Q1 masses (u)	80 ($^{80}Se^+$) or 31 ($^{31}P^+$)
Q3 masses (u)	96 ($^{80}Se^{16}O^+$) or 47 ($^{31}P^{16}O^+$)

To calculate the transport efficiency, the LGCQC5050 quality control material (AuNPs, LGC Standards Ltd., Teddington, UK) was used, with a numerical concentration specified in 1.47×10^{11} NPs/mL. A dilution was prepared in ultrapure H_2O to a final concentration around 30,000 NPs/mL. This suspension was measured by SP-ICP-MS, recording the signal for an m/z of 197 ($^{197}Au^+$), and using the same conditions specified in table S1. The final determination of the transport efficiency was carried out following the particle frequency method as described by Pace et al. [1]. Quantitative data on the Se mass present in the individual particles were then obtained by an external calibration of an aqueous Se standard.

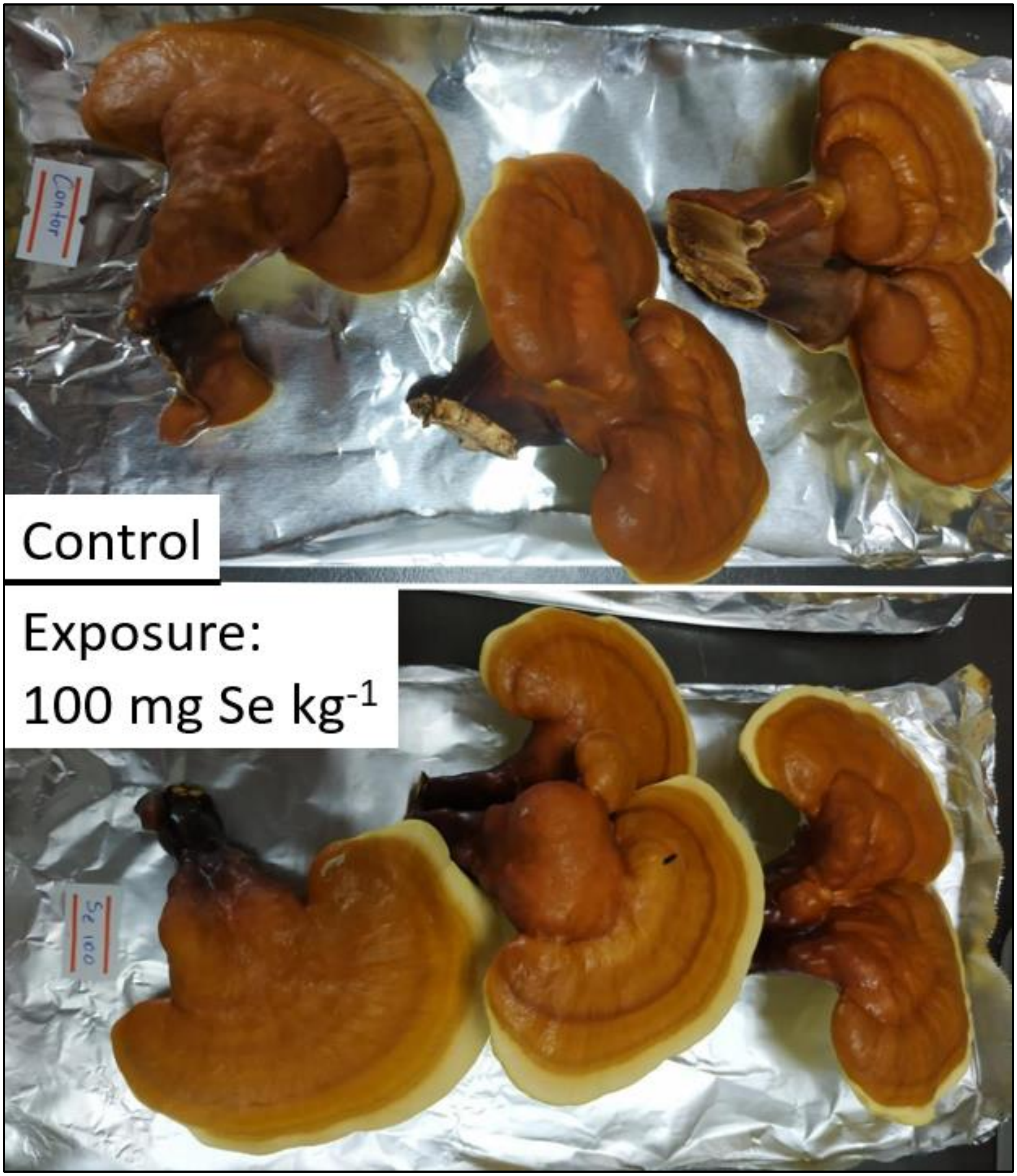

Figure S1: *G. lingzhi* fruiting body of control mushroom (top) and mushroom grown in the presence of 100 mg Se kg^{-1} substrate (bottom)

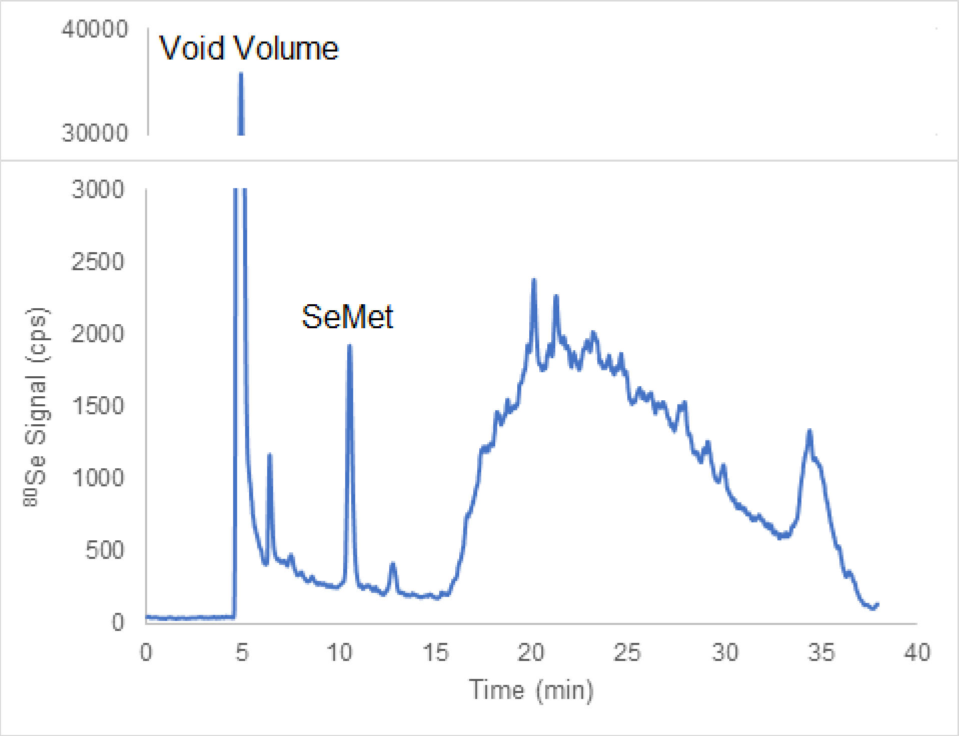

Figure S2: HPLC-ICP-MS chromatogram (C18 column) of a water extract from a sample of *Lingzhi* mycelium exposed to 20 mg Se kg^{-1} during growth

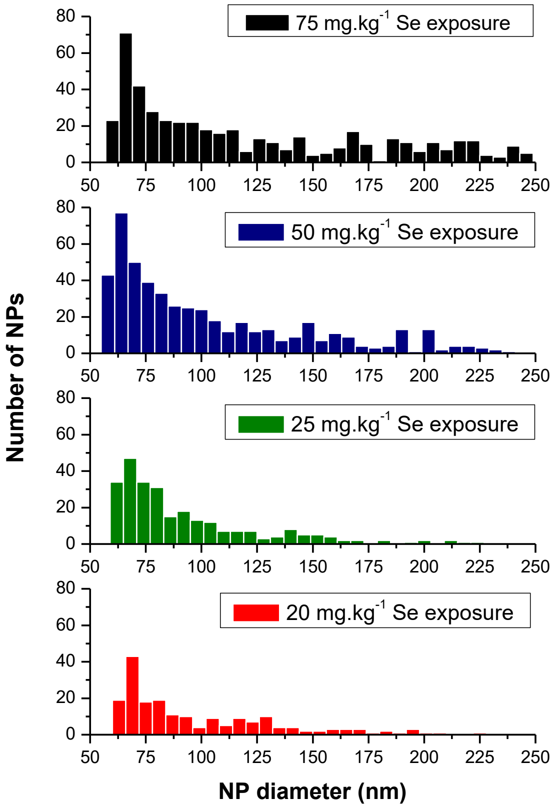

Figure S3: Histograms of the size distribution obtained by single particle ICP-MS in function of the concentration of selenite used for exposure (bin size: 6 nm)

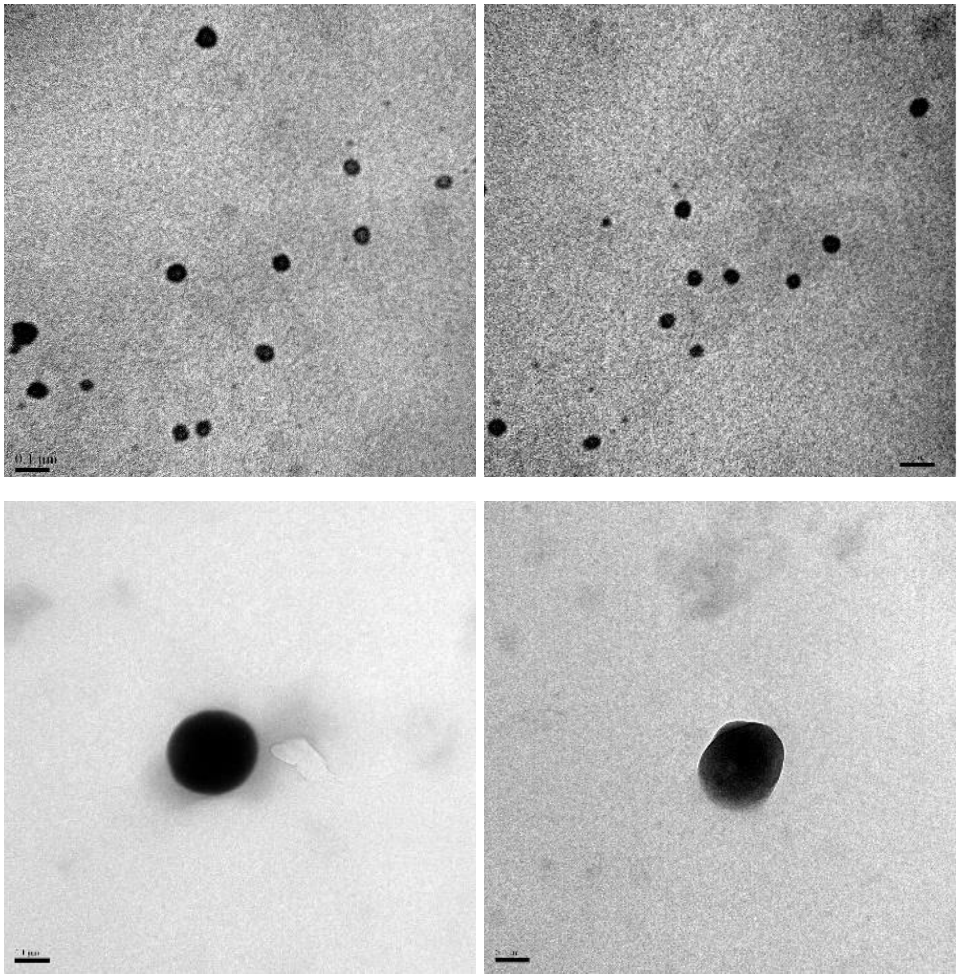

Figure S4: TEM images of a mycelium extract after exposure to 50 mg Se kg^{-1} as selenite (scale bar: 0.1 µm)

References for SI:

[1] H.E. Pace, N.J. Rogers, C. Larolimek, V.A. Coleman, C.P. Higgins, J.F. Ranville. Determining Transport Efficiency for the Purpose of Counting and Sizing Nanoparticles via Single Particle Inductively Coupled Plasma Mass Spectrometry. Anal Chem 2011, 83(24), 9361–9369. DOI: 10.1021/ac201952t
